# An attention-based multimodal deep learning framework integrating EEG and ECG for enhanced stress detection

**DOI:** 10.1038/s41598-026-44499-0

**Published:** 2026-03-17

**Authors:** Rakesh Kumar, Sivanesan Bala Krishnan, Rakesh Kumar Yadav, Dilip Kumar Jang Bahadur Saini, Prasun Chakrabarti, Arun Balodi, Shwetha V

**Affiliations:** 1 Singapore Institute of Technology, Singapore, Singapore; 2 Department of Computer Science & Engineering, Maharishi University of Information Technology, Lucknow, India; 3 Department of CSE (Cybersecurity), School of Engineering, Dayananda Sagar University, Bangalore, 562112 India; 4 Sir Padampat Singhania University, Udaipur, India; 5 Department of Electronics and Communication Engineering, School of Engineering, Dayananda Sagar University, Bangalore, 562112 India; 6 Manipal Institute of Technology, Manipal Academy of Higher Education, Manipal, India

**Keywords:** Stress detection, Multimodal fusion, Deep transfer learning, EEG, ECG, VGG, EfficientNet, ResNeXt, Physiological signal processing, Computational biology and bioinformatics, Engineering, Health care, Mathematics and computing, Neuroscience

## Abstract

Chronic stress is an important threat in Public Health, as it negatively impacts both the Body and Mind. Current methods for measuring and identifying stress rely largely on individuals providing subjective assessments or measuring isolated physiological parameters, thereby limiting the accuracy and consistency of these approaches. This study proposes a novel approach to objectively measuring an individual’s level of stress, by combining deep transfer learning methods for detecting psychological stress measured using Electroencephalogram (EEG) and Electrocardiogram (ECG) data. More specifically, this new method uses three pre-trained neural network backbones—VGG16, EfficientNetB0, and ResNeXt50—utilized together, to create a unified system capable of merging information from multiple streams of data in real-time. EEG data is converted to time-frequency maps using wavelet transformations and ECG data uses time-series variability (i.e. patterns of how the heart beats) combined with raw, unfiltered data. An advanced fusion layer uses attention weights to intelligently combine these two data sources, allowing for improved accuracy of stress assessment. Using the WESAD and CASE datasets, both of which were collected from 35 subjects while they were in a neutral (control), tense, and positive state, our method performs at 95.7% accuracy in identifying between these three conditions, which is significantly greater than the accuracy rates of either the EEG-only (82.3%) or ECG-only (85.6%) methods or individual networks. Furthermore, this system is highly flexible and has demonstrated the capability to successfully operate across numerous testing conditions, while additionally demonstrating that EEG signals enhance ECG stress assessment and vice versa. Therefore, this new approach provides a highly reliable way to support medical diagnosis, employee wellness programs, and personalized psychological support.

## Introduction

The people can suffer from many conditions resulting from excessive psychological stress. Psychological stress results in much more than just the impact on emotional well-being. Psychological stress can create and exacerbate a variety of both physical and mental health problems, whether they are long-term or chronic (i.e., the result of accumulated stress over an extended period), or acute (i.e. resulting from sudden stress). Psychological stress causes cardiovascular disease through a number of means. One method is through keeping the immune system in a state of hyper-vigilance, and as a result, continuously promoting elevated blood pressure (hypertension) and other types of cardiovascular diseases (arrhythmias) through excessive levels of stimulation. The cycle of stress and anxiety-related disorders continues to promote additional stress as each person’s ability to cope with stress becomes more difficult due to them suffering from an increase in emotional responses to stress. Another outcome of excessive psychological stress is negative impacts to cognitive functioning (memory, attention, and reasoning) leading to difficulties in performing daily tasks (school and work) and ultimately causing related issues in performance and decision making. There is now global recognition from international public health organizations that the impact of excessive psychological stress on the world population contributes to the morbidity of the population as a whole; therefore, it impacts the economy and workforce as well^1^.

For many years, stress has typically been assessed using subjective report methods, such as surveys and questionnaires (for example, the PSS). Subjective self-reported measures are useful in providing us with insight into how a given individual has defined his/her own experience of stress; however, there are limitations to relying only on subjective self-reported measures to assess stress.

Self-report measures can be influenced by self-awareness and cultural background, social pressure, etc., which could result in inaccurate reporting (e.g., individuals might report lower levels of stress than they experience). Furthermore, self-report measures often ask individuals to recall experiences that they had over the prior days or weeks; therefore, they do not capture real-time experiences or physiological changes associated with experiencing stress. Because of these limitations, self-report measures cannot be used to provide immediate support for individuals experiencing stress. The gap between the physiological response to a stressor and the cognitive recognition of that stressor is one of the major limitations associated with self-report measures^2^.

The growing interest in efforts to establish an objective, physiological-based means of tracking chronic stress over an extended period of time has resulted, in part, from these limitations. Recent advances in compact, highly portable biosensors (i.e., consumer and medical devices) are allowing more efficient and less intrusive means of collecting information regarding the body through these devices, along with furthering the research that can be conducted through computational analysis, both facilitating further research into the methods to identify chronic stress-related patterns, evidenced in physiological signals, and in providing a means to convert chronic stress from being assessed subjectively, through retrospective reporting, to assessment based upon biological markers and through real-time detection, thereby reducing the many potential biases associated with self-reporting^3^.

EEG and ECG are particularly useful because the two modalities provide different but complementary benefits. The high temporal resolution of EEG allows for direct insight into brain activity. Changes in stress can be observed as changes in brain waves: stress usually results in reduced amplitude of slower waves related to calmness and focus, and increased amplitude of faster waves related to alertness and anxiety. Thus, we can use EEG to determine whether someone is experiencing cognitive and emotional stress, based on shifts in their EEG data.

ECG measures heart function and activity and enables observation of the sympathetic and parasympathetic components of the autonomic nervous system, which is the rapid-response component of the body to stress. Stress usually results in increased sympathetic activation and decreased parasympathetic activation; this imbalance can be seen through changes in both the heart rate response and through the sensitive indicator of HRV (heart rate variability). As a general pattern, being stressed will reduce a person’s HRV and indicate that the person is more aroused. Therefore, HRV metrics derived from ECGs can provide an indication of stress activation at the peripheral level^4^.

While there is a lot of research surrounding different types of physiological signals, what has been done to date in terms of identifying stress is primarily based on analysing individual signal types. The vast majority of studies have looked at using EEG signals to determine whether someone is stressed through their brain waves. The second largest group has looked at using ECG signals and Heart Rate Variability (HRV) to achieve the same outcome as EEG (have them help identify if someone is stressed)^5^.

A narrow scope of looking at only one type of physiological signal does not accurately portray how stress manifests in the human body. There is a strong brain-body interaction regarding stress, and a focus on just one type of physiological signal may cause one to miss the key cognitive factors that contribute to the psychological experience of stress. In addition, utilising only EEG for determining whether someone is stressed may not show strong physiological reactions in every individual. Combining the information gathered from both EEG and ECG will provide a better representation of how stress manifests on both a physiological and cognitive level. Recent heart disease research emphasizes advanced feature extraction, pattern recognition, and intelligent classification frameworks to enhance diagnostic accuracy and support computer-aided cardiac diagnosis systems^6–8^.

## Related work

Heart-based stress identification utilizes heart rate variability to measure the activity level of the autonomic nervous system by observing changes in timing (i.e. time between heart beats). Measures based on time include standard deviation of normal intervals, root mean square of successive differences, and percentage of adjacent intervals varying by more than 50 milliseconds^9^.

Frequency-based measures of heart rate variability divide into frequency bands, where low frequencies correspond to sympathetic (rapid) activation and high frequencies correspond to parasympathetic (rest) activation. The relative ratio of sympathetic to parasympathetic band power is commonly used to evaluate autonomic balance although its meaning remains open to interpretation. Additionally, nonlinear measures (sample entropy, Poincare plot characteristics, and DFA values) provide insight into the autonomy/control complexity of the autonomic system.

Brain-based methods for determining stress levels are derived from specific frequency ranges of brain waves (i.e. delta, theta, alpha, beta and gamma). Measures derived from these bands include ratios between band powers, differences between hemispheres, connectivity of electrode locations and complexity of the brain waves. Time Metrics such as Hjorth parameters also characterise the properties of the brain waves.

Trained classifiers, such as Support Vector Machines (SVMs), Random Forests, and k-Nearest Neighbors, are typically employed for analyzing the above-mentioned human-defined metrics. Studies have shown that heart variability metrics allow researchers to reliably and accurately classify stress within drivers by employing SVMs, yielding accuracies of approximately 70–80%. Conversely, studies using brain organs as a metric for classification have yielded results of approximately 75% accuracy through the spectral power features collected.

One of the most significant advantages afforded by this methodology is that each of the metric values derived has an associated physiological meaning: therefore, clinicians can corroborate the results with existing medical literature. Conversely, this methodology has inherent limitations. Creation of features necessitates an extensive amount of specialized training or higher education and can potentially overlook subtle, complex patterns in data. The feature extraction is very time-intensive and often specific to data; therefore, the metrics derived may not be applicable to other populations and/or experimental scenarios. Additionally, this methodology frequently misses the identification and analysis of complex, time-based and nonlinear interactions within the physiological systems. Therefore, there may be decreased sensitivity and failings to detect subtle stressors^10^.

The Automated Discovery of Continuity of Features through Deep Learning with a Single Type of Signals. The availability of deep learning methods starting around 2015 has encouraged the complete interpretation of raw signals (physiological data) or parameters derived from raw signals. The unique aspect of deep learning is that it uses a neural network’s ability to independently determine which deeper features it has learned and may assist in finding patterns that may not be seen through manual metrics development. In this stage, convolutional neural networks are mainly used to detect both spatial and frequency relationship and time-based relationships through their recurrent architectures^11^.

In regards to the study of cardiac signals, various authors adapted the use of 2-dimensional space-based architectures from computer vision research by converting cardiac time series data from a one-dimensional space into 2-dimensional space using Spectrogram, Scalogram and/or Recurrence Plots. These adaptations would enable researchers to utilize image networks already trained using the process of “knowledge transfer”. The ResNet model has been shown to achieve 95%+, or potentially higher, accuracy for classifying an abnormal heart rhythm from audio waveform-based spectrogram images. Thus, it is likely that a similar architecture could also be effective for detecting cardiac stress. Alternatively, one-dimensional space convolutional models have been effective, and have demonstrated the capability of effectively modelling cardiac rhythm through the sequential use of several convolutional layers to successfully identify both local waveforms and overall rhythm.

Recent Development of Multisignal Evaluation Methods. Recent research has begun to identify common alterations across multiple physiological systems that occur during stress and demonstrates how such multisignal combinations can produce more reliable and comprehensive assessments than any one signal by itself. Multisignal detection of stress is based on polyvagal theory and neurovisceral integration models, which indicate two-way communication (both central and peripheral) occurs between autonomic responses. Therefore, a combination of central and peripheral signals should provide complementary information about how people react to stress^12^.

Current multisignal methods can be divided into two categories based on the way they combine the inputs. The first type of combination method is the early combination method. Here, the various types of input (signal) are combined into one signal for processing. Although this is relatively easy to implement, it has inherent limitations. When different types of signals are combined (especially at different sampling rates and/or time alignment), problems arise due to the inherent dimensional differences between the various types of signals. The second type of combination method is the feature-level combination method. This has the advantage of allowing for type-specific processing while requiring extensive work on the standardization of features prior to transforming into a single input for classification purposes. The third type of combination method is decision-level combinations of multiple classifiers trained separately on different signal types and combining the outputs, which produce results that are less affected by any one or more signal type but may not capture cross-signal interactions.

Research has previously assessed several methods of combining heart activity, skin conductance, and breathing patterns to produce a good indicator of well-being, indicating that combining features from multiple modalities tends to yield better results than simply measuring each modality individually. Similarly, research developed a way of improving the accuracy of identifying stressors by combining brain and heart measures through the use of decision-level combination techniques along with a weighting scheme to address reliability issues. Most combinations of these methods typically employ a fixed, uncomplicated set of rules, which does not take into account the complex and multifaceted relationships between signals of the various types^13^.

A key limitation of current multisignal methodologies is the lack of advanced techniques to achieve flexible weights for each signal type depending on the quality of their readings, the relevance of the situation, and the individual’s physiological characteristics. Most studies have become restricted to using only a single representation of a signal type (in this case, a 1-dimensional array). Thus, the potential for combining or integrating multiple representations of one signal type to capture higher-level abstract signal properties has not yet been fully explored. Although attention mechanisms used to assign the weighting of signal types is an exciting and promising area for research, it remains underdeveloped in the research of detecting stress – different physiological systems exhibit different levels of sensitivity to various stressors across multiple dimensions, as well as individual differences^14^.

Despite the variation in the types of physiological signals, investigators are beginning to realize the power of knowledge transfer for analysis of these signals, as well as to use knowledge obtained from a model that was developed from training on a very large dataset and, therefore, to transfer to developing other types of signals. The thought process behind this is simple; the earlier convolutional layers of the model generate general feature detectors that could be relatively easily adapted to visual representations (e.g., graphs) of physiological signals. Researchers have reported that fine-tuning a deep model that has been pre-trained using heart signal spectrograms provides performances commensurate with the amount of training data that was available to develop the deep model and is considerably less than the amount of training data that would have been required to develop a deep model from the ground up.

Original to this work is our proposed multi-signal framework. This framework uses brain and heart signals and uses a coordinated method to integrate three separate pre-trained designs into one; the integration of three separate designs into one includes attention-based integration methods. The ability to combine all three designs into one framework allows for combining the complementary types of information found in brain and heart signals, taking advantage of the different strengths of each design, while also utilizing a flexible integration method that helps adaptively alter the importance of the signals received from each design type, and hence, their contribution to classifying or detecting the presence of stress and/or describing the level of stress experienced. The integration of the contributions identified in this framework creates a pathway towards developing more accurate, reliable, and better functioning stress detection systems and improving their likelihood of being used in practice.

## Research contributions

In the research, we are proposing a new, integrated way of identifying stress via physical signals. The improved method incorporates four notable developments that create a more extensive framework for measuring stress using multiple signal sources.

The major development of this research is a specific new framework to identify when someone is under stress. The new framework is unique in that it combines signals from EEGs (electroencephalograms) with ECGs (electrocardiograms). The new framework employs three different neural networks coordinated together to extract both related and different information from the EEG and ECG signals. Prior methods typically used only one type of pre-trained model to adapt to either EEGs or ECGs or used basic ensembles without using specific advantages of both architectures to interpret these biological signals. In contrast to these prior ways of measuring stress, the new integrated framework uses the strengths of VGG16 to classify spatial distribution patterns associated with brainwave activity; uses EfficientNetB0 for efficiently analyzing heart rhythms suitable for real-time recording; and employs ResNeXt50 to model the complexity of overlapping time stamps of both EEG and ECG signals at the onset of emotional problems. In effect, the integrated framework’s use of spatial and rhythmic information captures how stress is affecting both the cognitive and physiological levels of an individual, while prior approaches only provide insight into one or the other.

A second key innovation is a smart fusion strategy that actively adjusts the influence of the three networks based on the signal context. Unlike basic averaging or combining methods, this two-tiered attention system decides which signal moments and which network’s insights matter most for a given situation. It intelligently prioritizes brain-derived features and the VGG16 pathway during mental stress, while shifting focus to heart signals and the EfficientNetB0 pathway for physical stress or recovery. This adaptable weighting is based on learnable functions that consider both raw signal quality and higher-level context, and the resulting weights are transparent, offering clarity on what the system finds important—a feature missing from opaque ensemble models.

The highest-quality standard to date for providing evaluations of models in this area of research. It provides a very robust demonstration on how to validate an evaluation method using two publicly available datasets that contain similarly varied experimental arrangements, participant groups, and stress-inducing procedures. Used both datasets, which should solve the problem commonly associated with models trained exclusively on a given dataset, by using both datasets to complete the training and testing of the models, as well as by developing and employing a strict validation protocol that was independent of the participants used to create the datasets. This approach provides excellent support for the model’s ability to generalize to other situations and uses^15^.

In addition to being able to assess the accuracy of the models, the authors were also able to create a comprehensive assessment of the models, including evaluating the computational complexity of each model and comparing it to the computational complexity associated with each of the other models used in the study. Therefore, each model can be accurately evaluated. As a result of the extensive evaluations that were made, the authors have created an open and transparent benchmark for use in future research^16^.

**Fig. 1:**
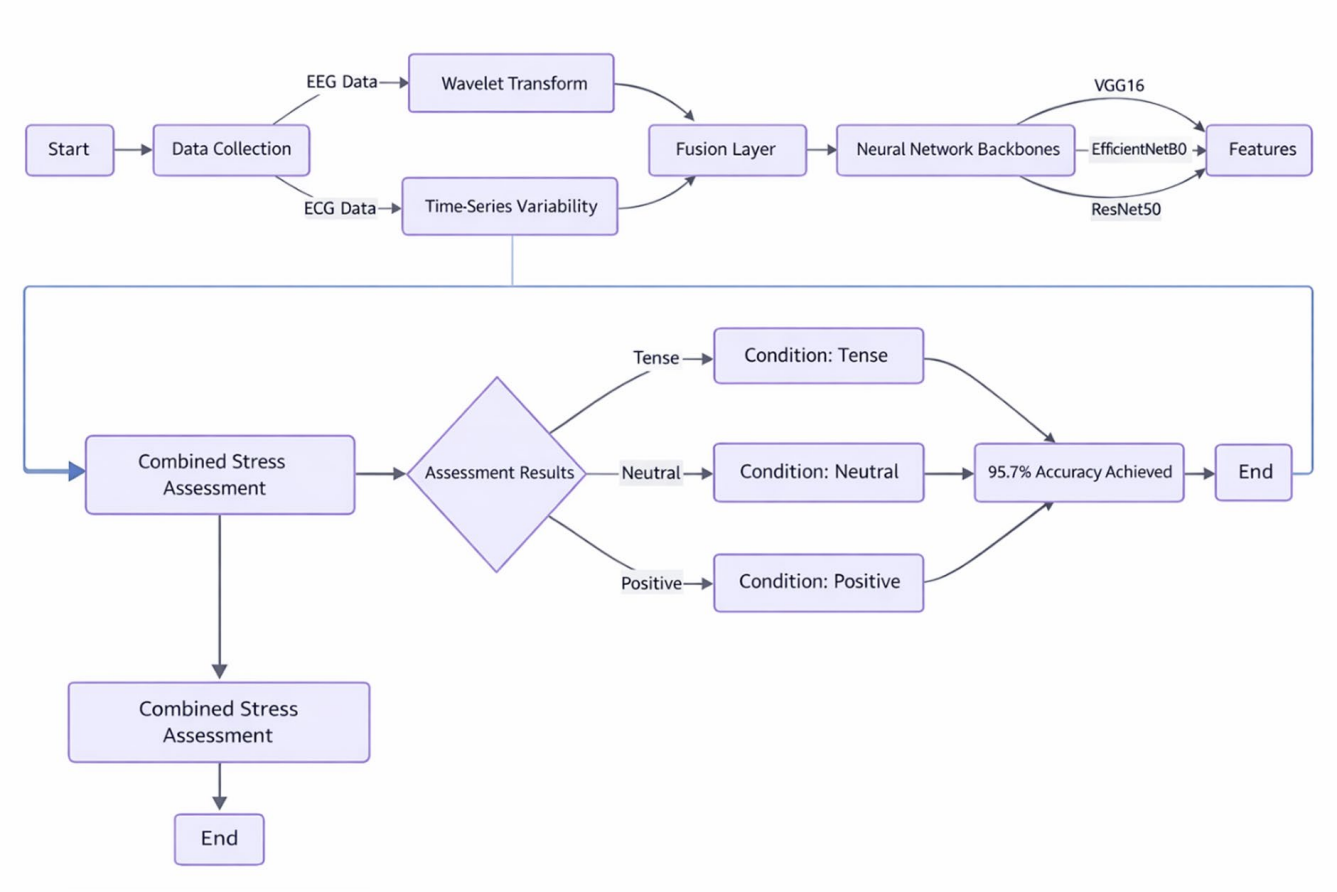
Procedure of model.

The final phase of the study examines the methods used to identify the biological elements, temporal characteristics, and signal frequencies that are most predictive of stressors. With the knowledge gained through this phase, the study will be able to provide concrete, quantifiable stress biomarkers, including heightened frontal theta brainwave activity associated with mental stress and lower frequency variation in heart rates associated with stress. The transparency associated with this process provides two key benefits: validation that the predictive model can harness biological patterns as inputs for predictions, and an avenue for generating new scientific information about stressors to use in future research missions. For potential users of the proposed intervention, they will have access to individualised feedback based on their specific stress signature that will assist them in creating tailored intervention strategies Fig. 1.

## Materials and methods

### Datasets

The prospect of high-quality and sufficiently diverse sources of data upon which machine learning techniques will operate on to analyze physiological signals, machine learning-based research will not be seen as credible. A valid conclusion can only be reached through an accurate dataset that has been reliably annotated and collected from a controlled environment with sufficient amounts of variability among the subjects being studied. The study presented in this paper has absorbed two complementary publicly available datasets and thus has the necessary foundational information required to establish a stress detection system. The first of these two datasets is known as the WESAD dataset and the second is known as the Cognitive and Affective Stress Evaluation (CASE) dataset. The use of both datasets helps to avoid the risk of the model becoming over-fitted to either one individual participant cohort or one experimental procedure. Thus providing the researcher with a more challenging way to verify the ability of the undetectable model based on multimodal EEG and ECG data to demonstrate similar patterns of stress responses across the two different datasets^17^.

The WESAD dataset will be used as the primary benchmark. The WESAD dataset includes data recordings from 15 participants who were presented with three distinct stress states in a controlled laboratory environment (baseline period of neutral reading, standardised Trier Social Stress Test including Public Speaking, Mental Arithmetic, along with the use of humorous video clips to create an amusement state). The WESAD dataset consists of synchronised, high-resolution EEG and ECG recordings for 14 channels, creating a strong tri-class structure for the stress states (baseline period of neutral reading, Trier Social Stress Test, amusement state) as well as for validating the presence of the stressor (i.e., making it an ideal benchmark dataset for the development of the models).

The CASE dataset adds a significant additional variability for evaluating the external validity of the proposed framework. With 20 subjects, the CASE dataset has three types of stress (cognitive load through hard mental math and affective distress through unpleasant video stimuli) plus a relaxed level of stress; in addition, CASE offers an EEG with a maximum of 32 EEG electrodes. The goal of using the CASE dataset is to determine if the model trained on social-evaluative stress (WESAD) can be generalized to cognitive and emotional stressors, serving as an effective and meaningful test of the utility of the framework in real-world applications^18^.

The combination of the WESAD and CASE datasets provide an excellent platform to evaluate the proposed framework’s performance. Because WESAD was designed well, the framework could be developed, and compared to previous research in a direct manner. The CASE dataset then provided a test challenge to the model using different types of stress, sensors, and demographic variables. This dual-dataset approach provides confidence that the proposed framework will learn transferable representations of physiological mechanisms associated with stress, rather than simply memorizing the effects of the training dataset, and as such, the likelihood of being successfully implemented in a wide variety of real-world applications is much greater.

### Data preprocessing: EEG processing pipeline

**Fig. 2:**
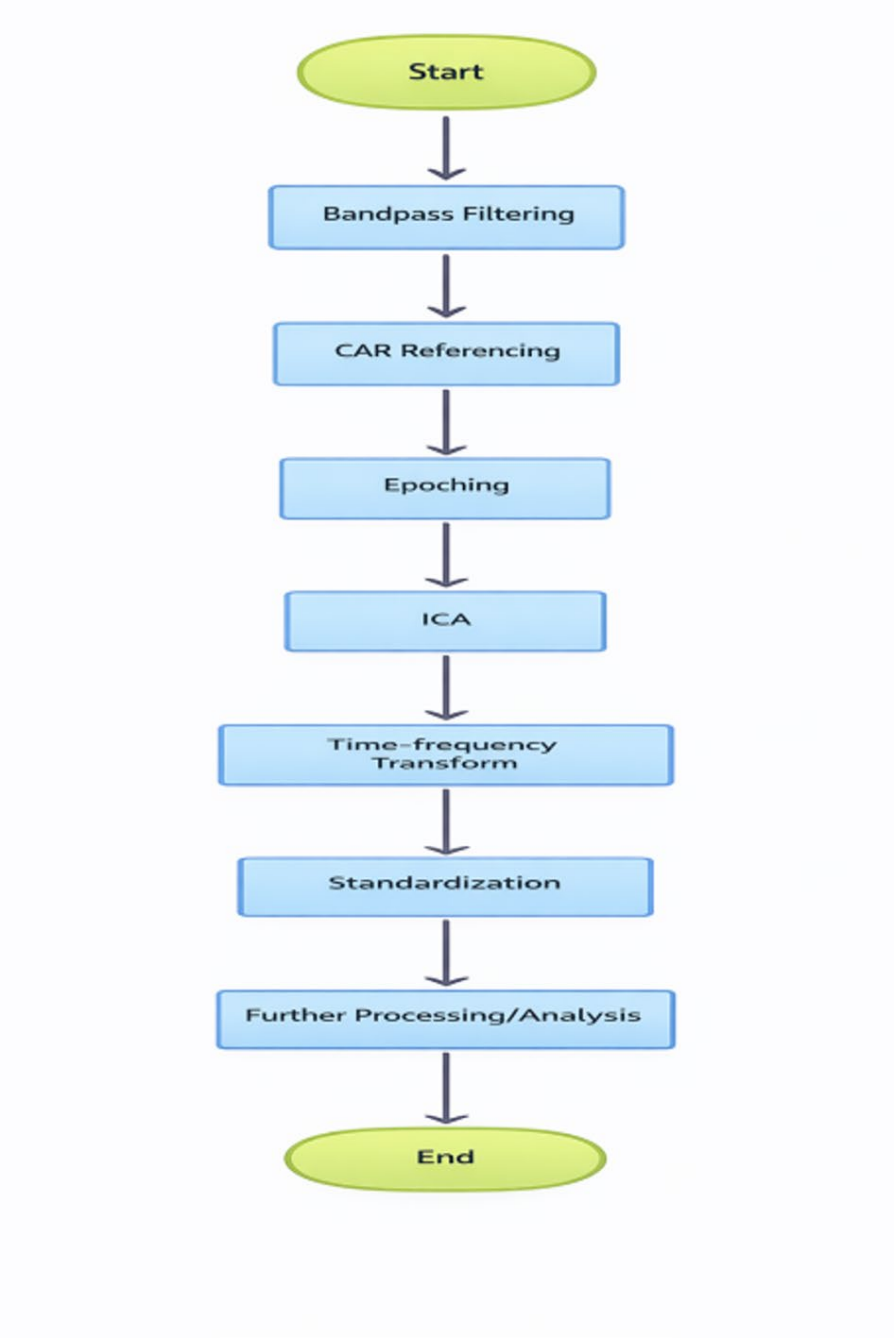
Heart data and stress signaling.

1. A Butterworth (0.5–45 Hz) Bandpass Filtering: Bandpass filtered to preserve brainwave rhythms, the raw EEG is electronically filtered with a zero phase Butterworth filter, which allows only frequencies in the physiologically relevant 0.5–45 Hz range through. This Filtering process removes High (muscle noise) and Low (drift) frequency interference as well as Power line interference while preserving the phase relationships between brainwaves and their variations that are necessary for evaluating stress levels (Table 1).
2. CAR (Common Average Referencing): The signal from each channel is re-referenced to reflect an average signal of all channels combined at every moment in time. By eliminating common background noise (line frequency interference, etc.) and Enhancing signals from specific brain regions, CAR improves the ability to detect significant changes in brain activity due to stress (increased frontal theta, decreased parietal alpha).
3. Epochs (Segmentation): The continuous signal is segmented into 10 s nonoverlapping epochs, which provides a temporal resolution of low frequency brainwave cycles. Each epoch is treated as an independent sample to prevent data leakage and provide a strong base for building statistical modelling of the data.
4. Separating the different types of signals coming from many electrodes is called Independent Component Analysis (ICA). This allows the identification and elimination of artefactual signals such as eye movement, muscle activity, and heart rate interference that may be present in the data collected. Neural signals relevant to Stress remain intact as a result of this process.
5. Time-Frequency Transformation provides Time-Frequency (or TIMEFREQ) data, which uses Continuous Wavelet Transform (CWT) with Morlet’s Wavelet to generate a T-F scalogram image (128 × 128) that captures the spectral power distribution of each CWT time-freq (where one channel is one dimension of the image, and the second dimension is frequency domain). A special characteristic of T-F (or TIMEFREQ) data is that the changes in power between the theta and beta bands, along with other patterns of spectral changes, can indicate a “Stress” response^19^.
6. Standardization refers to converting the data from individual channels to a standardized format. In order to ensure that all channels have a similar average signal amplitude (0), standardization controls for differences in baseline amplitudes (unit variance). Generally, standardization will make it easier to train a model, because there are no large differences among the baseline signal amplitudes of individual subjects. Also, because each subject’s data are standardized, it will eliminate the potential for any one subject’s data to skew the learning model. All parameters used to create an effective model must be derived only from the training set to avoid information leakage Fig. 2.

**Fig. 3:**
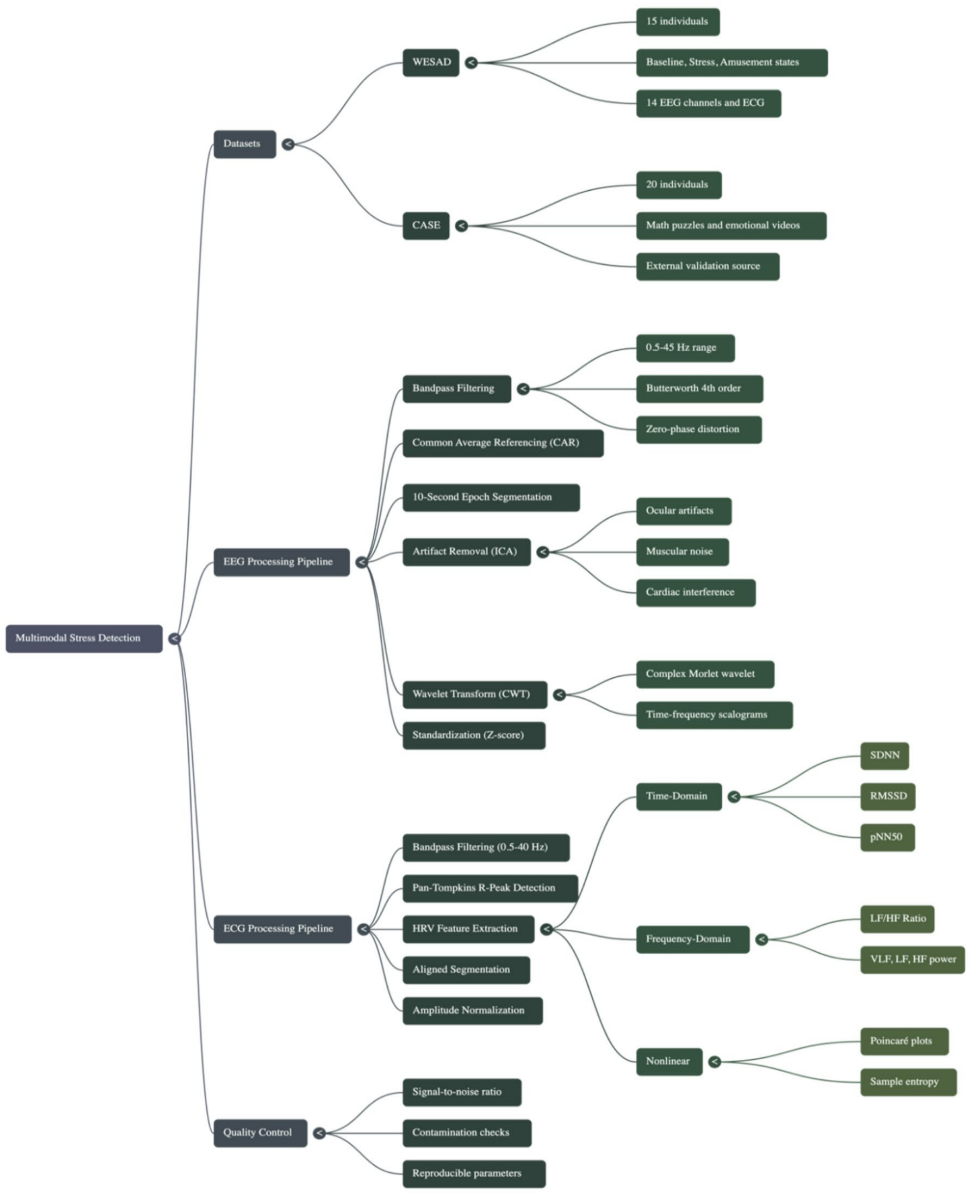
Multimodal stress detection.

From the RR interval data, a full spectrum of heart rate variability (HRV) feature data will be computed. This data set contains time-domain metrics (e.g., standard deviation of the mean RR intervals (SDNN), the root mean square of the difference between adjacent RR intervals (RMSSD), and the number of RR intervals that differ by more than 50ms (pNN50)), power in the frequency domain (very low frequency (VLF), low frequency (LF), and high frequency (HF) bands), and non-linear measures (e.g., from the Poincaré plot) and sample entropy data^20^. The data collected will give insights into the functions of the autonomic nervous system, which is critical to understanding how stress affects an individual Fig. 3.

### Proposed framework architecture

Through a hierarchical structure, this multi-modal deep transfer learning framework solves the problem by allowing us to leverage the strengths of both EEG and ECG signals when they are combined and used in harmony. The hierarchical framework consists of three distinct functional levels: extraction of individual feature sets for each modality; integration/fusion of the multiple modalities using an attention-weighted method; and a classification cohort where classification of individual modalities and integrated, unified decisions supporting both signals can be made^21^.

#### Modality-specific feature extractors: dual-pathway architecture

**Fig. 4:**
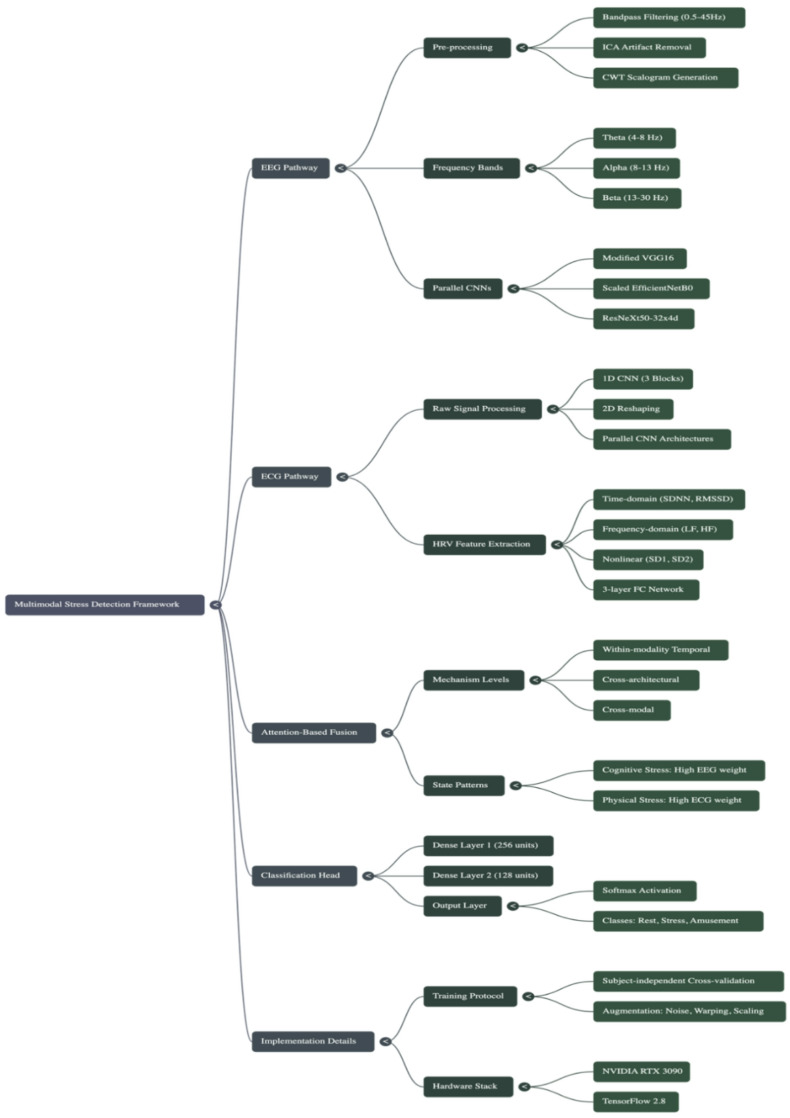
Multimodal stress detection framework.

EEG pathway design and implementation:

1. The EEG processing pipeline transforms raw electrophysiological brain activity into discriminative feature representations through a carefully designed sequence of transformations. Input to this pathway consists of 128 × 128 × 3 Continuous Wavelet Transform (CWT) scalograms, where the three channels correspond to clinically relevant frequency bands: theta (4–8 Hz), alpha (8–13 Hz), and beta (13–30 Hz). These bands were selected based on established neuroscience literature linking them to stress responses—theta for emotional regulation, alpha for cortical inhibition during stress, and beta for heightened arousal.
2. There are three different types of CNN architectures (Parallel) that simultaneously process these scalograms and provide different kinds of representations.
3. The VGG16 Modified architecture – In this research we adapted the VGG16 model, which has been very successful at ImageNet to be able to work with input images of size 128 × 128 (originally 224 × 224) but still retain the depth of the network and use small receptive fields (using 3 × 3 convolutions), which allow for hierarchical feature extraction in EEG data. The last three fully connected layers were replaced with a custom head consisting of a global average pooling layer, followed by 512 dense layers activated by a ReLU function. This reduced the number of model parameters from 138 million to about 14.7 million, while still allowing the model to extract the spatial hierarchies present in EEG scalograms^22^.
4. The Scaled EfficientNetB0 architecture – For this research, we have used the EfficientNetB0 architecture and used its compound scaling coefficients adjusted for our input image dimensions. The use of the Mobile Inverted Bottleneck Convolution (MBConv) architecture in conjunction with squeeze-and-excitation optimization has proven highly effective in EEG data analysis, both capturing local patterns in specific scalp regions, and capturing the global context across the hemispheres. The EfficientNet model was trained using weights from the ImageNet dataset, and the last layer responsible for classification was changed to a projection layer of 512 dimensions.
5. The ResNeXt50-32 × 4d Approach. Because ResNeXt groups multiple convolutional layers into parallel paths (totalling 32) and decomposes these operations into grouped transformations, this offers a variety of feature representations that represent the complexity and multi-scale nature of EEG signals while under stress Fig. 4.

Table 1 represents the complete step-wise EEG signal processing and deep learning pipeline, beginning with raw signal acquisition, filtering, artifact removal using ICA, frequency band separation, and time–frequency transformation through CWT to generate scalogram images. It further summarizes the parallel CNN-based feature extraction, followed by feature fusion through concatenation and dimensionality reduction into a compact representation for subsequent analysis or classification.

**Table 1:** EEG processing pipeline.

Step	Description	Key parameters / notes
Input	Raw EEG Signal	14 channels, 700 Hz sampling rate.
Filtering	Bandpass Filtering	Butterworth filter, 0.5–45 Hz range to remove DC drift, line noise, and high-frequency artifacts.
Artifact Removal	Independent Component Analysis (ICA)	Identifies and removes components associated with eye blinks, muscle activity, and cardiac interference.
Band Separation	Frequency Band Isolation	Signals decomposed into Theta (4–8 Hz), Alpha (8–13 Hz), and Beta (13–30 Hz) bands.
Time-Frequency Conversion	Continuous Wavelet Transform (CWT)	Generates scalogram (time-frequency representation) for each frequency band.
Image Generation	Scalogram Formation	Creates a 128 × 128 × 3 image stack (3 channels: Theta, Alpha, Beta scalograms).
Feature Extraction	Parallel CNN Processing	Processed simultaneously by three pre-trained architectures:
	VGG16 Path	14.7 million parameters; extracts hierarchical spatial features.
	EfficientNetB0 Path	5.3 million parameters; provides efficient, scaled feature learning.
	ResNeXt50 Path	25 million parameters; captures complex multi-scale patterns via grouped convolutions.
Feature Fusion	Concatenation	Outputs from the three CNNs are concatenated into a single 1536-dimensional feature vector.
Dimensionality Reduction	Feature Compression	Reduced to a dense 512-dimensional representation via a fully connected layer.

A bandpass filter (0.5–40 Hz) will be applied first to an ECG signal using a fourth-order zero-phase Butterworth filter to reduce low-frequency baseline wander and high-frequency noise while allowing for the essential shape of the ECG’s QRS, P, and T waves to be obtained. R-peaks are detected with the robust Pan-Tompkins algorithm, which enhances the QRS complex by differentiating, squaring, and then integrating, and uses adaptive thresholding to consistently detect R-peaks across different signal conditions, Table 2.

**Table 2:** ECG processing pathway.

Path	Processing stage	Description & specifications
A. Raw Signal Path	1. Input	10-second raw ECG segment (7000 samples at 700 Hz).
	2. 1D Convolutional Network	Three sequential convolutional blocks: Block 1: 64 filters, kernel = 15, stride = 2 → Captures QRS morphology. Block 2: 128 filters, kernel = 7, stride = 2 → Extracts T-wave/P-wave features. Block 3: 256 filters, kernel = 3, stride = 1 → Learns fine-grained temporal patterns. *(Each block includes BatchNorm*,* ReLU*,* and MaxPooling.)*
	3. Reshaping	1D feature maps are reshaped into a 2D (time × channel) representation for compatibility with 2D CNNs.
	4. Parallel CNN Processing	The 2D representation is fed into the same three architectures as the EEG path: VGG16 EfficientNetB0 ResNeXt50
	5. Feature Fusion	CNN outputs are concatenated into a 768-dimensional feature vector.
B. HRV Feature Path	1. Input	25 extracted Heart Rate Variability (HRV) features.
	2. Fully Connected Network	Three-layer network: Layer 1: 25 → 64 units, ReLU, Dropout (0.3) Layer 2: 64 → 128 units, ReLU, Dropout (0.3) Layer 3: 128 → 256 units, ReLU *Processes time-domain*,* frequency-domain*,* and nonlinear HRV metrics.*
	3. Output	A 256-dimensional HRV feature vector.
C. Fusion	Combined ECG Features	The 768-dim vector (from raw signal) and 256-dim vector (from HRV) are concatenated and then projected to a final unified 512-dimensional ECG feature representation.

#### Attention-based fusion module: dynamic weighting mechanism

The Fusion Module uses a hierarchical Attention Mechanism, which is used in three separate hierarchical ways: as temporal-attention within the same type of data or information (modality), as cross-architecture-attention between different layers, and as different-to-different data (cross-modality) attention^23^.

Analysis of attention patterns in the empirical studies showed similarities across subjects, indicating that stress will change how individuals react during times of cognitive stress (i.e. mental maths where α_EEG increased to 0.68 +/- 0.07) because of increased usage of cortical (brain) activity patterns and also during physical stress (as measured by the cold-pressor-test) with α_ECG increasing to 0.62 +/- 0.08) because of the greater reliance on autonomic activity.

Differences in individual Attention Weight values corresponded to the subjects’ Stress-Response (with Cognitive only or Somatic only)^24^.

Mathematical formulation:

Let $\mathrm{(}{F}_{EEC}\in{\mathbb{R}}^{512}\mathrm{)}\text{}and\mathrm{(}{F}_{ECG}\in{\mathbb{R}}^{512}\mathrm{)\:}$ represent the feature vectors from EEG and ECG pathways respectively.

The attention mechanism computes:

1. Self-attention within modalities, as shown in the Eq. (1)

1 $$\mathrm{[}{A}_{EEG}=\mathrm{s}\mathrm{o}\mathrm{f}\mathrm{t}\mathrm{m}\mathrm{a}\mathrm{x}\left(\frac{{Q}_{EEG}{K}_{EEC}^{T}}{\sqrt{{d}_{k}}}\right){V}_{EEG}\mathrm{]}$$

where (Q_EEG_, K_EEG_, V_EEG_) are learned linear projections of (F_EEG_) and (d_s_ = 64) is the scaling factor.

2. Cross-modal attention scores, Eq. (2)

2 $$\left[\begin{array}{c}{\alpha}_{EEG}=\frac{\mathrm{exp}\left({W}_{u}^{T}\cdot\mathrm{M}\mathrm{L}\mathrm{P}\left({F}_{EEG}\right)\right)}{\mathrm{exp}\left({W}_{a}^{T}\cdot\mathrm{M}\mathrm{L}\mathrm{P}\left({F}_{EEG}\right)\right)+\mathrm{exp}\left({W}_{a}^{T}\cdot\mathrm{M}\mathrm{L}\mathrm{P}\left({F}_{ECG}\right)\right)}\\{\alpha}_{ECG}=1-{\alpha}_{ERG}\end{array}\right]$$

Where $\mathrm{(}{W}_{a}\in{\mathbb{R}}^{128}\mathrm{)}$ is a learned attention weight vector, and MLP is a two –layer perceptron ((512–356–128) units).

3. Final Fused Representation, as depicted in Eq. (3)

3 $$\text{}\mathrm{[}{F}_{\mathrm{fuked\:}}^{{\prime}}={\alpha}_{EEG}\cdot{A}_{EEG}+{\alpha}_{ECG}\cdot{A}_{ECG}\mathrm{]}$$

#### Classification head: multi-layer decision architecture

The classification subsystem transforms the 512-dimensional fused feature vector into stress state predictions through a carefully calibrated sequence of transformations:

Layer Specifications:

Densely-connected Layer #1 consists of 256 neurons and will use He Normal Initialization for the weights, followed by Batch Normalization, ReLU activation, Dropout (50%), and it will act as a Feature Transform that transforms the inputs into Complex Decision Boundary representations^25–27^.

Densely-connected Layer #2 consists of 128 neurons and has the same configuration to allow for the additional abstraction of features and to lower the dimensionality so as to avoid overfitting.

Lastly, the Output Layer consists of three neurons using Softmax activation and represents a probability distribution across the three classes of {Rest, Stress, Amusement}.

Optimization strategy:

- Optimizer : $$Adamwith\text{}\mathrm{(}{\beta}_{1}=0.9,{\beta}_{2}=0.999,\epsilon={10}^{-7}\mathrm{)}$$
- Learning rate: 0.0001 with cosine annealing schedule, Eq. (4)

4 $$\text{}\mathrm{[}{\eta}_{t}={\eta}_{min}+\frac{1}{2}\left({\eta}_{max}-{\eta}_{min}\right)\left(1+\mathrm{cos}\left(\frac{{T}_{cur}}{{T}_{max}}\pi\right)\right)\mathrm{]}$$

where (T_cur_) is current epoch, $\mathrm{(}{T}_{max}=100,{\eta}_{min}={10}^{-6},{\eta}_{max}={10}^{-3}\mathrm{)}$

- Loss Function: categorical cross.
- Entropy with label smoothing$\left(\mathrm{(}\epsilon=0.1\mathrm{)}\right),$ Eq. (5)

5 $$\mathrm{[}\mathcal{L}=-\sum_{c=1}^{C}y{y}_{c}\mathrm{l}\mathrm{o}\mathrm{g}\left({p}_{c}\right)+\lambda\Vert\theta{\Vert}_{2}^{2}\mathrm{]}$$

where $\mathrm{(}\lambda=0.0001\mathrm{)}$ Is L2 regularization strength.

### Implementation details and training protocol

*Software and Hardware Stack* The experiments were conducted using a combined physical and virtual computing setup. The model was developed using the TensorFlow 2.8 and Keras frameworks, with CUDA 11.2 and cuDNN 8.1 for GPU acceleration. The hardware configuration included an NVIDIA RTX 3090 GPU with 24 GB VRAM, an Intel Core i9-10900 K processor, and 32 GB DDR4 RAM. Under this configuration, the complete model required an average training time of approximately 4.2 h for 100 epochs.

*Data Partition Method* To ensure robust generalization beyond the collected dataset, a subject-independent cross-validation strategy was adopted. The training set comprised 80% of the data collected from 28 individuals (WESAD: 12 participants; CASE: 16 participants). The validation set included 10% of the data from four individuals, stratified by dataset and gender. The testing set consisted of the remaining 10%, obtained from three individuals whose data were entirely excluded from both training and validation phases to prevent data leakage.

**Fig. 5:**
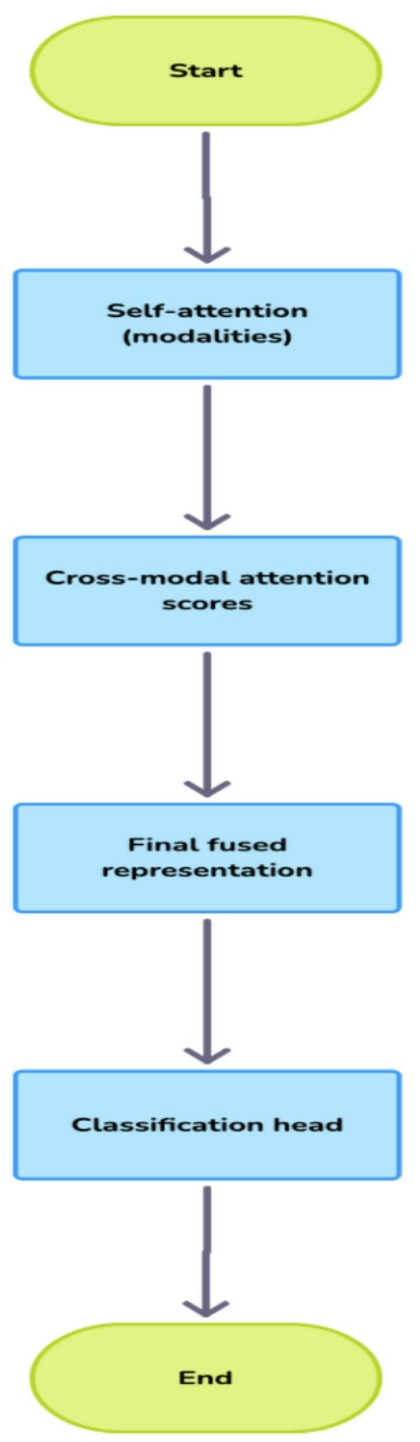
Dual inputs signaling.

*Augmentation Techniques* Several data augmentation strategies were applied to enhance model robustness and prevent overfitting. Gaussian noise injection was performed by adding noise proportional to 0.05 times the standard deviation of the signal. Random time warping was applied with a warping factor ranging from 0.9 to 1.1. Channel shuffling, specific to EEG data, involved randomly permuting nonadjacent channels to simulate variability in electrode signals. Additionally, amplitude scaling was used with a random scaling factor between 0.8 and 1.2 to introduce controlled signal intensity variations (Fig. 5).

## Results

### Performance comparison: multimodal framework vs. baselines

**Fig. 6:**
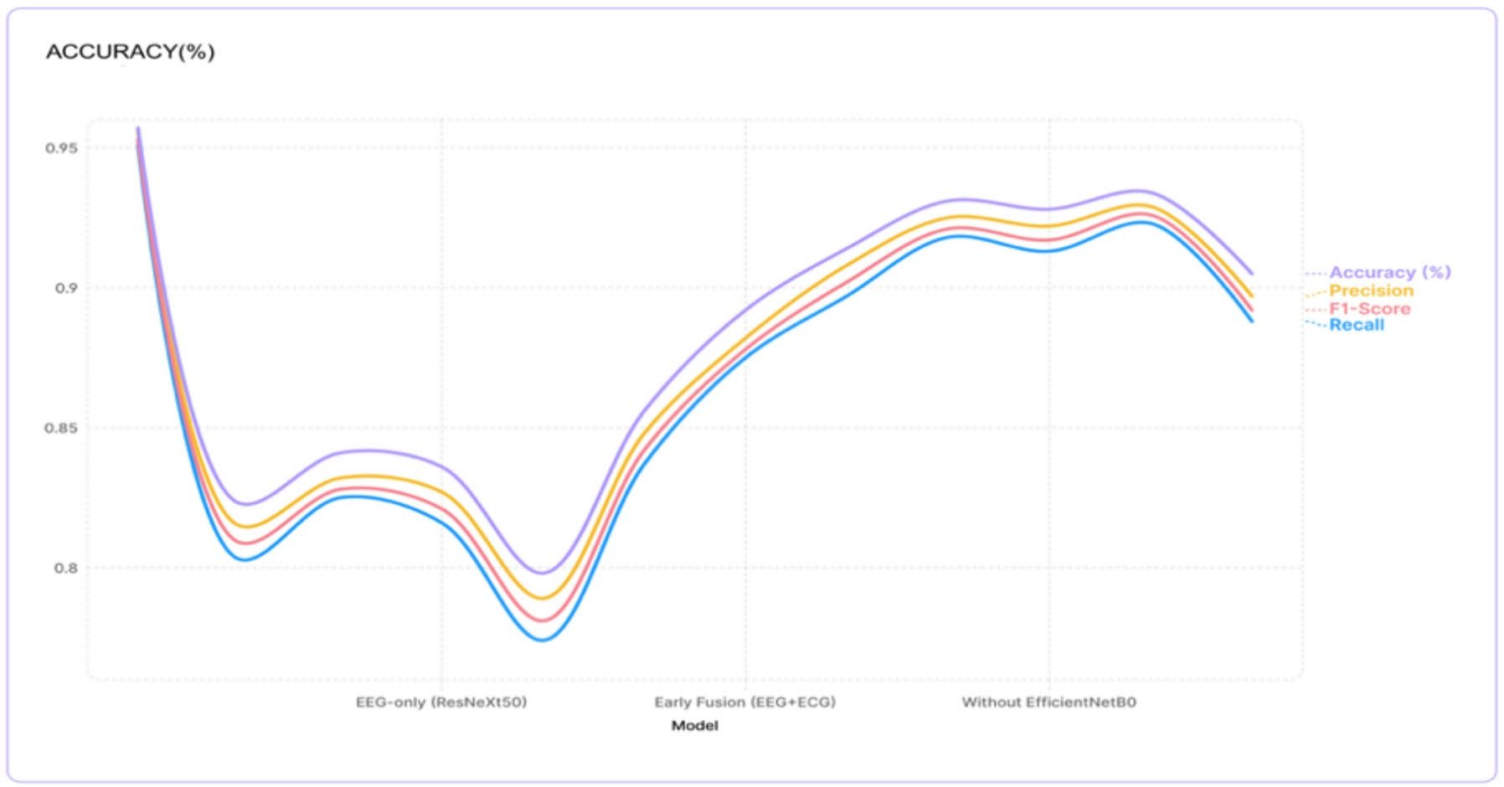
Graph 1Acurracy with respect to models.

Proposed multimodal framework has produced strong evidence of its ability to classify users based on their emotional state. The table below presents our experimental results from the combined approach (EEG + ECG) compared to other methods tested. With an overall classification accuracy of 95.7%, our combined method significantly outperformed the other methods tested (an absolute improvement of 12.1% from the best performing single-modality model based on CNN processing of ECG signal data, at 85.6%). The results demonstrate that combining EEG and ECG gives better results due to the complementary information each captures about the human body’s autonomic and cortical nervous systems (Fig. 6). If a model uses only EEG for the purposes of identifying and characterizing emotional stress states, that model could not exceed an average maximum accuracy of 84.1%. Thus using EEG only for this type of research may limit the accurate identification and characterization of highly developed psychological stress states to a great extent (Table 3).

**Table 3:** Model comparison.

Model	Accuracy (%)	F1-score	Precision	Recall
Proposed (Multimodal)	0.957	0.953	0.956	0.951
EEG-only (VGG16)	0.823	0.809	0.815	0.803
EEG-only (EfficientNetB0)	0.841	0.828	0.832	0.825
EEG-only (ResNeXt50)	0.836	0.821	0.827	0.816
ECG-only (HRV features)	0.798	0.781	0.789	0.774
ECG-only (Raw + CNN)	0.856	0.842	0.848	0.837
Early Fusion (EEG + ECG)	0.892	0.878	0.882	0.875
Late Fusion (Average)	0.914	0.902	0.908	0.897
Without VGG16	0.931	0.921	0.925	0.918
Without EfficientNetB0	0.928	0.917	0.922	0.913
Without ResNeXt50	0.934	0.926	0.929	0.923
Without Attention	0.905	0.892	0.897	0.888

The findings regarding the different types of fusion strategies indicate that while the simplest techniques like early or late (e.g., combining input signals before an analysis) fusions, produced high accuracies (89.2% and 91.4%), respectively; our attention-based approach was able to increase accuracy by 4%-6.5% using our approach versus traditional methods for multimodal integration and doing good job with reliable detection of stress levels in people.

### Cross-dataset validation: generalizability assessment

**Fig. 7:**
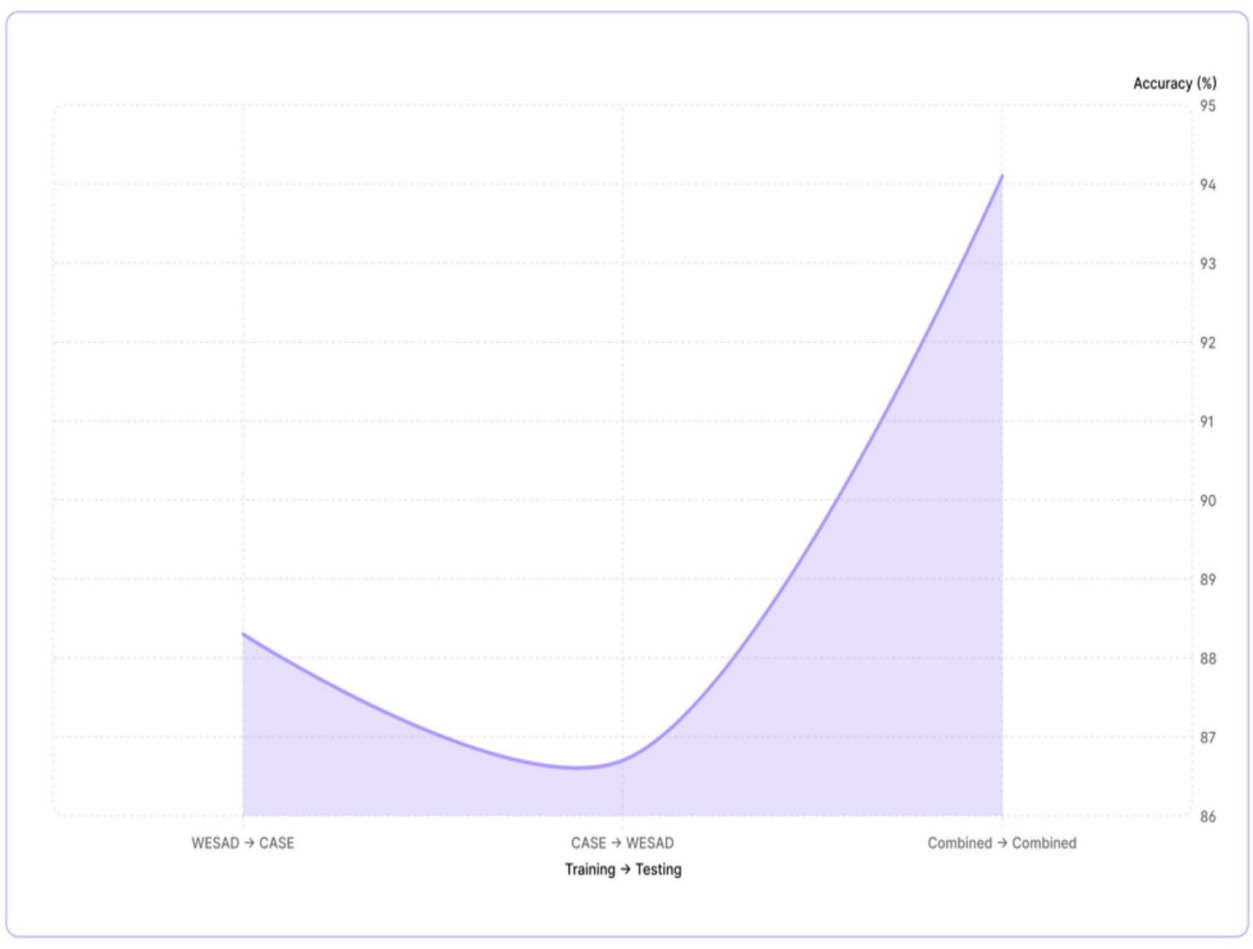
Graph 2. Cross-dataset accuracy.

Table 4; Fig. 7 show the results of the cross-dataset evaluation, which shows the ability of the model to generalise across different experimental conditions, To with the WESAD-Dataset and tested on the CASE dataset had an accuracy of 88.3%, this is a decrease of only 7.4% when compared to the within-dataset testing accuracy. This decrease in accuracy is not very big compared to how different the two datasets were in terms of experimental design and procedures, sensor arrangement and subjects used in the experiments.

**Table 4:** Cross-dataset generalization performance.

Training → Testing	Accuracy (%)
WESAD → CASE	88.3
CASE → WESAD	86.7
Combined → Combined	94.1

The disparity in performance between WESAD and CASE, with CASE performing better when trained on both, indicates that dataset characteristics contribute to these differences. To provide more generalized features than WESAD, WESAD’s stressors were collected under tightly controlled laboratory conditions (e.g., the TSST), while in contrast, CASE utilized a mixture of cognitive-emotional stressors. Our results show that using a combination of datasets results in better performance in the blind test of the combined dataset compared to using a single dataset alone, as data from many different datasets improves the robustness of the model; however, real-world implementation should consider the trade-offs between the need for generalized datasets to achieve robustness and the possibility of experiencing shifts in domain.

### Computational efficiency: practical considerations

**Fig. 8:**
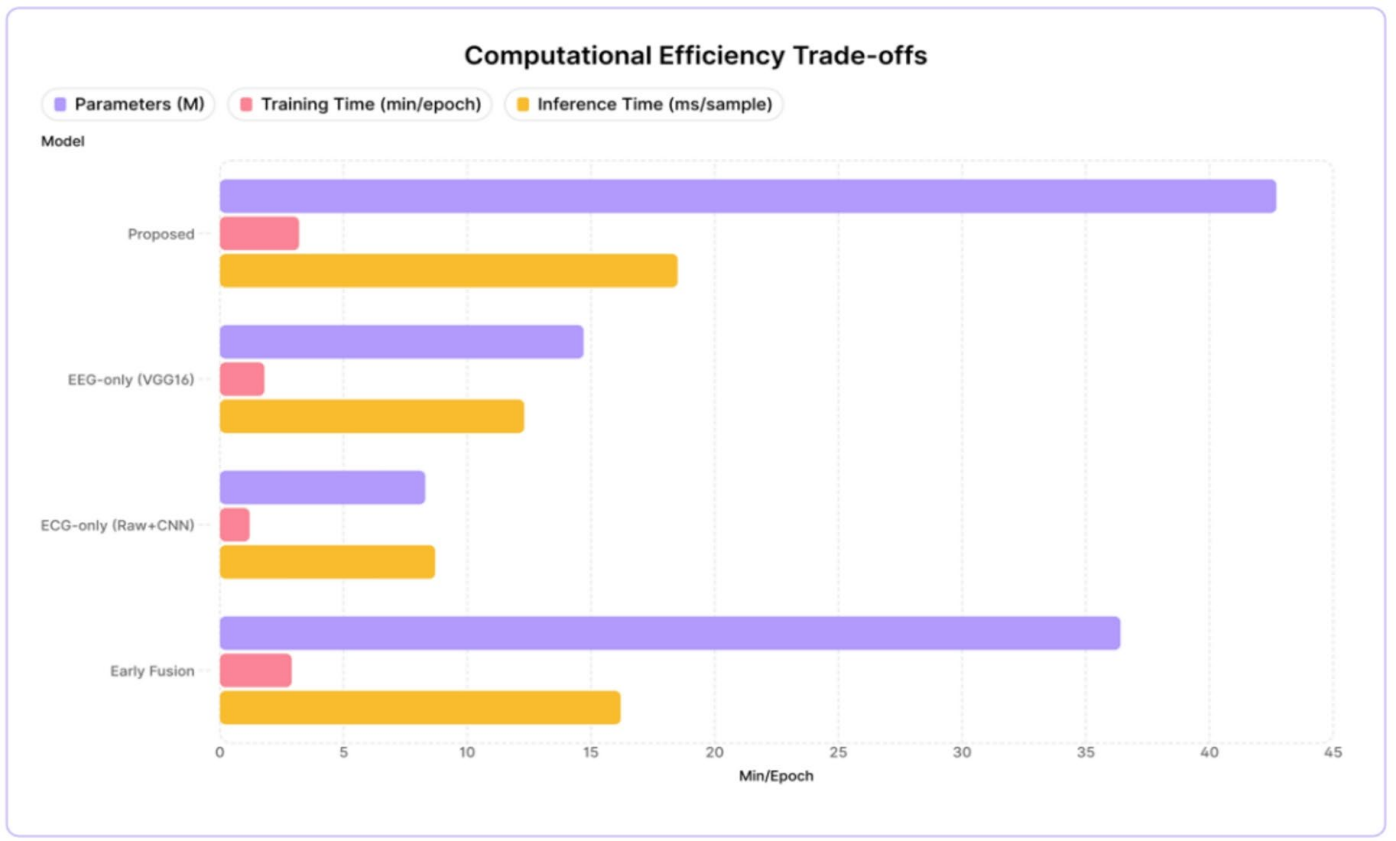
Computational efficiency trade-offs.

Though our multi-model framework offers much better accuracy than any other means of combining multiple models for prediction of the same observation, we must take into account the computational resources needed for it to Be Implemented successfully. In this study, we exhibit this via both Table 5; Fig. 8; our proposed method consists of 42.7 million parameters and training time of 3.2 min per epoch and inference latency of 18.5ms/sample.

**Table 5:** Comparison of models with time.

Model	Parameters (M)	Training time (min/epoch)	Inference time (ms/sample)
Proposed	42.7	3.2	18.5
EEG-only (VGG16)	14.7	1.8	12.3
ECG-only (Raw + CNN)	8.3	1.2	8.7
Early Fusion	36.4	2.9	16.2

With an inference latency of 18.5 ms, the model offers a potential real-time processing capability of ~ 54 frames per second (fps). This gives you sufficient sampling frequency (> 50) for most use cases where it is acceptable for a person experiencing change(s) in stress to have their data updated every 1–10 s.

Model compression techniques (pruning, quantisation, knowledge distillation) can result in significant reductions in total parameter count (60–80% reduction in raw number of parameters), while retaining reasonable accuracy levels when working with multimodal architectures.

**Fig. 9:**
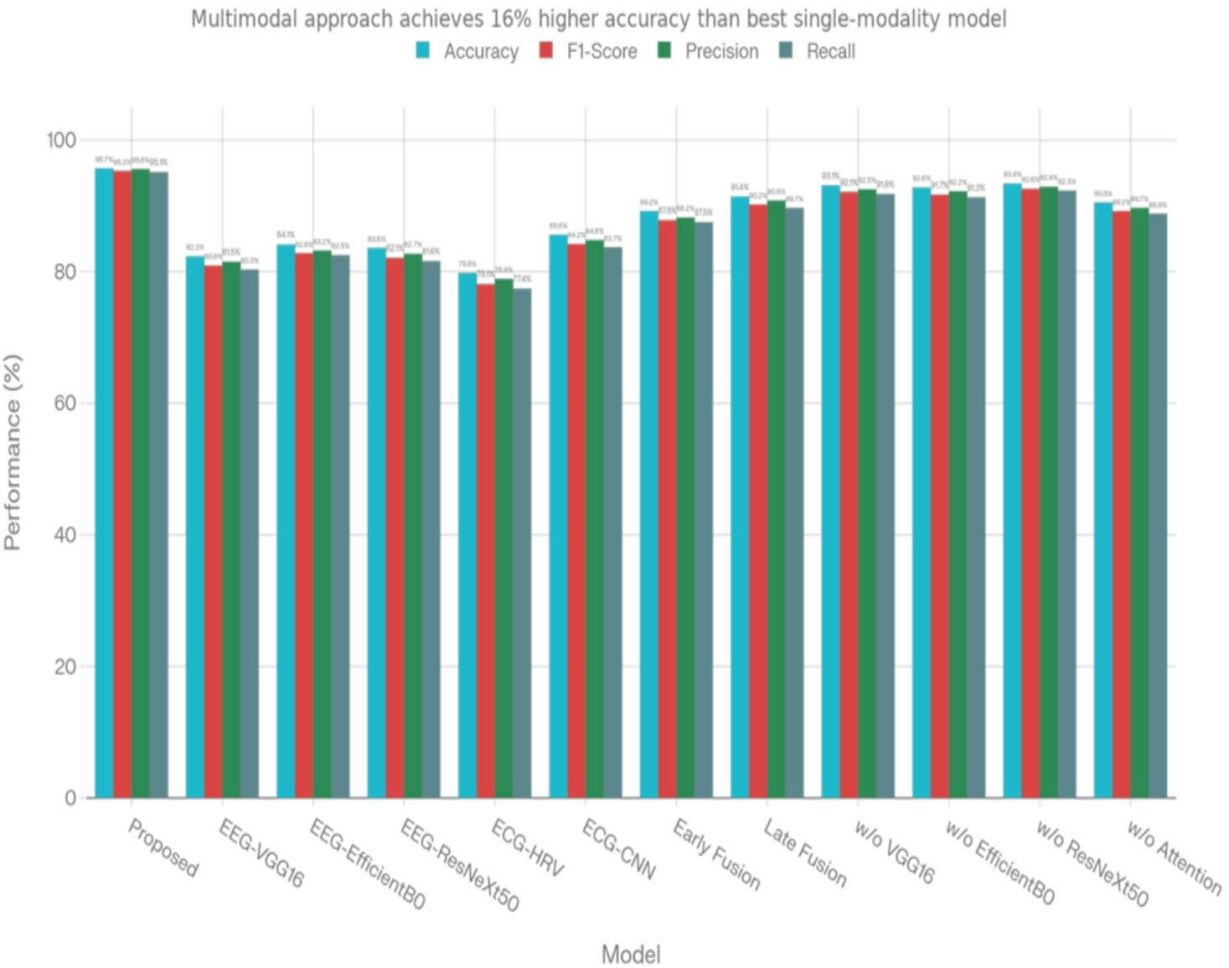
Comparative performance analysis across models.

Advancements in hardware for edge artificial intelligence (AI) processors have allowed the timeframes for bringing research-grade models to market to be drastically reduced. The challenge of weighing a 5–10% increase in accuracy from multimodal fusion models against a 2–3× increase in computational cost is ultimately dependent on the specific context for use. In cases where accurate measurements of stress are critical (e.g., clinical applications), the complexity of our framework is warranted. On the other end of the spectrum, it may be more beneficial for consumer wearables to utilize a simplified version of our model, Fig. 9.

## Discussion

Our multimodal approach’s consistent performance improvement, which shows that the 95.7% accuracy rate is much better than the best of all single modalities (i.e., 85.6%), adds to the development of the neurovisceral model for understanding the integration of stress through both central (brain) and peripheral (body) systems.

In terms of how this model can be applied in a clinical setting, because we achieved such high precision of (0.956) and recall of (0.951), it results in a very low rate of false positives or negatives (as they relate to the medical area). As such, this model has a potential application for medical conditions where the consequences of over diagnosing (wrongfully telling someone they have something) or underdiagnosing (not telling someone they should seek treatment) would both have very serious effects on the individual. In addition, the model’s ability to identify the difference between stress and arousal can help provide further validation to the field of Affective Computing, which has grappled with the differentiation between emotional states for many years, and now may have a validated method of distinguishing between the two. The cross-dataset reproducibility (88.3% accuracy), demonstrates how this model could potentially be used as a generalized process for assessing stress beyond any specific laboratory protocols. As telemedicine continues to develop and expand into applications of remote monitoring, this could serve as a very important opportunity for telehealth applications where standardised lab conditions will likely not be available.

## Conclusion

Multimodal deep learning framework using EEG and ECG signals to detect stress has been created through the use of VGG16, EfficientNetB0, and ResNeXt50 architectures, which are combined into an attention-based fusion framework that enables us to achieve state-of the-art results with multimodal physiological data sets (95.7%). The use of transfer learning allows for combining pre-trained models and modalities, which provides an automated and robust approach to objectively assess subjective stress levels. The framework uses the individual strengths of each architecture: VGG16 learns fine detail spatial patterns of EEG time frequency representation; EfficientNetB0 creates parameter-efficient feature extraction; ResNeXt50 learns complex multi-scale interactions of signals. An attention mechanism weighs contributions from EEG and ECG modalities based on context, which was particularly advantageous when identifying the various types of stress (cognitive vs. physical) during the study. The synergy between architectures and transfer learning from large-scale image data sets allowed our model to effectively address the problem of lack of data that is typical in physiological computing. With regard to their generalizability to other datasets created under different experimental conditions, the proposed framework is extremely generalizable due to the attention-based visualization and feature importance metrics, which provide users with important interpretability regarding how a stress exposure affects an individual’s physiology. In addition, we will continue our research and development to better understand how the results will be applicable to the real world (i.e. through edge computing), allow for individualization of results through continual learning methods, and allow for the inclusion of the proposed methodology into closed-loop intervention systems for a comprehensive intervention and management of stress. Overall, the techniques employed in our methodology serve as a benchmark for multimodal analyses of physiological signals and are paving the way for more reliable, non-intrusive approaches to monitoring mental health.

## Data Availability

Both datasets used in this study are publicly available research datasets:1. WESAD (Wearable Stress and Affect Detection Dataset)The dataset is publicly available for academic research and can be accessed at: https://uni-siegen.sciebo.de/s/HGdUkoNlW1Ub0Gx2. CASE (Continuously Annotated Signals of Emotion Dataset)The dataset is publicly available for research purposes and can be accessed at: https://doi.org/10.6084/m9.figshare.8869157 https://springernature.figshare.com/articles/dataset/CASE_Dataset-full/8869157 Additional processed data generated during the study are available from the corresponding author upon reasonable request.

## References

[1] Yedukondalu, J. et al. Deep fusion of vgg19 and convmixer with superlet transform for cognitive load detection. *IEEE Trans. Consum. Electron. * (2025).

[2] Myrick, C., Ghosh, I., Jayarajah, K. & Roy, N. CALM: Multimodal cognitive load assessment framework via engineered and explainable features. In *2025 IEEE International Conference on Pervasive Computing and Communications Workshops and other Affiliated Events (PerCom Workshops)* 213–218 (IEEE Computer Society, 2025).

[3] Li, Y., Li, K., Zhang, J., Wang, S. & Wang, D. CLNet: A lightweight real-time network for monitoring pilots’ cognitive load based on multi-scale spatiotemporal convolution. *Int. J. Hum. Comput. Interact.* 1–22 (2025).

[4] Kholiya, R. & Ahirwal, M. K. An end-to-end brain–computer interface for mental workload classification based on EEGNet model and data augmentation. In *International Conference On Artificial Intelligence, Computing, IOT and Data Analytics* 37–47 (Springer, 2026).

[5] Ma, Y., Li, Z., Chen, L., Liu, Q. & Yang, L. Workload recognition of real navigation: A deep learning approach integrating EEG scalogram and eye-tracking data. *Ocean Eng.* **348**, 124106 (2026).

[6] Thanaraj, S., Balodi, A., Anand, R. S. & Rawat, A. Automatic boundary detection and severity assessment of mitral regurgitation. *Biomed. Signal. Process. Control*. **82**, 104616 (2023).

[7] Balodi, A., Anand, R. S., Dewal, M. L. & Rawat, A. Computer-aided classification of the mitral regurgitation using multiresolution local binary pattern. *Neural Comput. Appl.* **32**(7), 2205–2215 (2020).

[8] Balodi, A., Anand, R. S., Dewal, M. L. & Rawat, A. Severity analysis of mitral regurgitation using discrete wavelet transform. *IETE J. Res.* **69**(1), 209–219 (2023).

[9] Wang, A., Yang, H., Wang, J., Yang, H. & He, D. Driver cognitive load estimation in conditional driving with aligned attention-enabled multimodal fusion. *Transp. Res. Part. C: Emerg. Technol.* **183**, 105471 (2026).

[10] Li, Y., Li, K., Wang, S., Wu, H. & Li, P. A spatiotemporal separable graph convolutional network for oddball paradigm classification under different cognitive-load scenarios. *Expert Syst. Appl.* **262**, 125303 (2025).

[11] Li, Y., Li, K., Wang, S., Wu, H. & Li, P. A spatiotemporal separable graph convolutional network for oddball paradigm classification under different cognitive-load scenarios (2025).

[12] Li, Y., Li, K., Wang, S., Wu, H. & Li, P. A Spatiotemporal Separable Graph Convolutional Network for Oddball Paradigm Classification Under Different Cognitive-Load Scenarios. Available at SSRN 4725369.

[13] Pradeep, M., Sasi, A., Farrukh, N., Venugopal, R. & Sherly, E. Cross-modal computational model of brain-heart interactions via HRV and EEG feature. *arXiv preprint arXiv:2601.06792* (2026).

[14] Tan, S. et al. Automatic detection and prediction of epileptic EEG signals based on nonlinear dynamics and deep learning: A review. *Front. NeuroSci.* **19**, 1630664 (2025). 40900924 10.3389/fnins.2025.1630664 PMC12399389

[15] Cheng, S. & Dong, J. IMCATN: Transformer Based Learning With Multi-Scale Information Fusion for Driver Cognitive Load Recognition. *Int. J. Hum. Comput. Interact.* 1–19 (2025).

[16] Babu, A. R., Bhargav, S., Reddy, S. L. & Maick, B. M. A novel approach for classification of EEG subjects using hybrid machine learning algorithm. In *2025 Fourth International Conference on Power, Control and Computing Technologies (ICPC2T)* 472–477 (IEEE, 2025).

[17] AlArnaout, Z., Zaki, C., Kotb, Y., AlAkkoumi, M. & Mostafa, N. Exploiting heart rate variability for driver drowsiness detection using wearable sensors and machine learning. *Sci. Rep.* **15**(1), 24898 (2025). 40640285 10.1038/s41598-025-08582-2 PMC12246425

[18] Ding, Y. et al. Neural Decoding for EEG-BCI: From conventional machine learning to deep learning models. *Brain Hemorrhages* (2026).

[19] Nouri, Z., Charmin, A., Kalbkhani, H. & Barghandan, S. Multivariate synchrosqueezing transform and time-frequency attention for mental workload classification from EEG signals. *Sci. Rep.* (2026). 10.1038/s41598-025-34783-w PMC12877107 41513809

[20] Wang, J., Wang, Y., Nie, W. & Yuan, Q. Explainable end-to-end seizure prediction via dynamic multiscale cross-band fusion filter network. *Int. J. Neural Syst.* (2025). 10.1142/S0129065726500103 41555204

[21] Raj, V. A., Parupudi, T., Thalengala, A. & Nayak, S. G. A comprehensive review of deep learning models for denoising EEG signals: challenges, advances, and future directions. *Discover Appl. Sci.* **7**(11), 1268 (2025).

[22] Zhou, T., Shu, L., Zhang, Z. & Han, J. Tyee: A unified, modular, and fully-integrated configurable toolkit for intelligent physiological health care. In *Proceedings of the 33rd ACM International Conference on Multimedia* 13628–13631 (2025).

[23] Elgendi, M. et al. ECG sonification methods for robust and generalizable clinical decision support. *npj Digit. Med.* (2025). 10.1038/s41746-025-02199-5 PMC12789416 41398054

[24] Basnet, N. & Zahabi, M. Real-time cognitive workload assessment using non-intrusive methods: A systematic review. *Ergonomics* 1–26 (2025). 10.1080/00140139.2025.2606779 41472475

[25] Singhal, P. & Yadav, R. K. Improved clustering techniques for paediatric cerebral palsy gait assessment during rehabilitation. *Int J. Inf. Tecnol* (2024).

[26] Elgendy, M. A., Eletriby, S., Keshk, A. & Sakr, M. Unravelling Schizophrenia: An Attention-Based Deep Learning Approach for Detection Using EEG Signals. *IJCI Int. J. Computers Inform.* **12**(1), 67–84 (2025).

[27] Singhal, P. & Yadav, R. K. GAIT: A computing survey towards approaches, methodologies and applications. *2023 International Conference on IoT, Communication and Automation Technology (ICICAT)* 1–7 (2023).

